# Seven Cases of Aphonia

**Published:** 1864-01

**Authors:** W. H. Butler

**Affiliations:** A. Asst. Surgeon U. S. A.


ART. II.—*Seven Cases of Aphonia, collected by* W. H. BUTLER, *A. Asst. Surgeon U. S. A.*

I have collected seven cases of Aphonia, resulting generally from cold. The cases I have seen have generally been well in other particulars. The cases have been recorded as the patients were accidentally met in the various wards of the hospital; in six the cause seems traceable to colds; the seventh is charged to a previous attack of measles. The average of the six recorded ages is 24 years.

The following well authenticated case of recovery of the voice is interesting: A soldier who had been suffering from aphonia for some time, and finally transferred to the Invalid Corps for this disability, while passing down Seventh street in this city, accidentally fell into a cellar-way and was considerably bruised, but on gaining the side-walk he found compensation for all his pains in the entire restoration of his powers of speech. No doubt the fall had dislodged some thickened membrane from about the vocal cords.

*Case of Aphonia, No.* 1.—George Dunbar, aged 23, born in Michigan, enlisted August, 1862; for some time has had a hacking cough after hard, labor; has been subject to fever and ague from childhood up to three years ago; thinks he has had it every year. Was on duty three months after enlistment, about November 15th, had ague, took cold, had cough and loss of voice followed; cough continued two weeks, and in three weeks completely lost his voice. Has done only light duty from November 15th to January 15th, 1863, when he had what he supposes was typhoid fever; was admitted in Armory Square Hospital February 8th; had had diarrhœa for two weeks before admission, and it was persistent up to the date of his discharge, March, 1863. Have heard nothing of him since.

*No.* 2.—George Perry, aged 24, private Co. D, 20th Michigan Volunteers; enlisted August, 1862; was then well and hearty; of sanguine nervous temperament; was well until October, 1862, when he had intermittent fever, which kept him in hospital ten days, and was debilitated for two weeks after; before recovering from that, or at about three weeks from the first attack of fever, he was taken with inflammation of the lungs; was sick in hospital three weeks; while recovering from the latter disease thinks he got up too quick, caught a cold and lost his voice; coughed up blood after this, several times. Is now, July 18, 1863, well, but cannot speak above a whisper. July 25, 1863, is well and hearty; speaks in a husky whisper; thinks his voice is weaker now than three months ago. The uvula and muscles arching from it seem emaciated, and look rather pale. The tonsils look about natural.

*No.* 3.—Private Thomas Yagle, aged 30, sanguine nervous temperament, enlisted August. 1862; was then in good health; kept well until December, 1862, when he caught a heavy cold; had sore throat for a month with sore chest, and a dry, hacking cough; as he recovered from the cold he suddenly lost his voice, and yet speaks in a whisper; he also has a dry cough as if something irritated his throat. The tonsils and uvula seem emaciated, and have a pale look. (Case recorded July 22, 1863; feels well; patient had had typhoid fever in June, 1861, but was entirely over its effects when he enlisted in the following August.)

*No.* 4.—James D. Stearns, private 7th Co. U. S. Sharp Shooters, enlisted September, 1862, in good health; is of sanguine nervous temperament kept well for six months, then he had a slow fever, called by the doctors "putrid fever;" sore throat was associated with it; the latter affection produced hoarseness and affected the voice. This trouble lasted five weeks; was well after this until May 1st, 1863, when he caught a hard cold from exposure on picket duty, which required him to lay on the ground. He recovered from this July 14th; caught cold again and has been unable since to speak above a whisper—well otherwise.

*No.* 5.—S. B. Labar, aged 27, private Co. D, 15th N. J.; previously healthy; never took a dose of medicine till he entered the army; January 17th was attacked with a severe cold; began to lose his voice, and on the 20th could not speak loud; was sent to camp hospital, White Oak Church, Stafford county, Va., where he staid two weeks, then returned to his tent, but did no duty till in March; has lost flesh, and continues to, up to this time, October 9, 1863. The throat on examination looks red in places, but not marked. He complains of some pain in left side of chest.

*No.* 6.—John A. Barr, aged 23, Co. H, 34th Massachusetts, enlisted July, 1863, was well and doing duty at Fort Lyon, Va., until January, 1862, when he had measles; while recovering from this disease he gradually lost his voice; entered Armory Square Hospital July 10th, rather debilitated, but able to do light duty; can only talk in a whisper. A month since, there was a time, say a week, when his voice returned in a slight degree, but soon relapsed.

*No.* 7.—Henry Wright, aged 22, 2d Batt. Inv. Corps, 49th Co., entered the service November, 1861, was well and hearty then, and kept well until about November 1st, 1862, when having been sent on picket, he was compelled to lay for two hours in the wet, and caught a severe cold, followed by expectoration of blood and inflammation of the lungs; was sick four weeks, and has been an inmate of hospital since, having entered Armory Square Hospital in June, then to Convalescent Camp, and now he is on duty here. November 4th, is pretty well, complains of shortness of breath; has occasional neuralgic pains through left lung; has some cough, raises white, thickphlegm; has slightly enlarged tonsils, and a reddish blush about arch of palate; says he has had a raw feeling about the throat since he had the inflammation of lungs. For some time during his illness he was very hoarse, and gradually afterward lost his voice. He now speaks with some effort in a moderate whisper.

*Armory Square Hospital,*

*Washington, Dec. 10th,* 1863.

---

THE operation for ligature of the subclavian artery, performed in November by Dr. Armsby, of Albany, has been successful. The ligature came away on the twenty-ninth day. It is now (Dec. 28th) forty days since the operation, and the patient is able to attend to business.—*Boston Med. Jour.*

